# Identification of Metastasis-Associated Biomarkers in Synovial Sarcoma Using Bioinformatics Analysis

**DOI:** 10.3389/fgene.2020.530892

**Published:** 2020-09-11

**Authors:** Yan Song, Xiaoli Liu, Fang Wang, Xiaoying Wang, Guanghui Cheng, Changliang Peng

**Affiliations:** ^1^Department of Nephrology, The Second Hospital, Cheeloo College of Medicine, Shandong University, Jinan, China; ^2^Department of Hematology, The Second Hospital, Cheeloo College of Medicine, Shandong University, Jinan, China; ^3^Institute of Medical Sciences, The Second Hospital, Cheeloo College of Medicine, Shandong University, Jinan, China; ^4^Department of Pathology, The Second Hospital, Cheeloo College of Medicine, Shandong University, Jinan, China; ^5^Central Research Laboratory, The Second Hospital, Cheeloo College of Medicine, Shandong University, Jinan, China; ^6^Department of Orthopedics, The Second Hospital, Cheeloo College of Medicine, Shandong University, Jinan, China

**Keywords:** synovial sarcoma (SS), bioinformatics analysis, differentially expressed genes (DEGs), protein-protein interaction (PPI), hub genes, survival analysis

## Abstract

Synovial sarcoma (SS) is a highly aggressive soft tissue tumor with high risk of local recurrence and metastasis. However, the mechanisms underlying SS metastasis are still largely unclear. The purpose of this study is to screen metastasis-associated biomarkers in SS by integrated bioinformatics analysis. Two mRNA datasets (GSE40018 and GSE40021) were selected to analyze the differentially expressed genes (DEGs). Using the Database for Annotation, Visualization and Integrated Discovery (DAVID) and gene set enrichment analysis (GSEA), functional and pathway enrichment analyses were performed for DEGs. Then, the protein-protein interaction (PPI) network was constructed via the Search Tool for the Retrieval of Interacting Genes (STRING) database. The module analysis of the PPI network and hub genes validation were performed using Cytoscape software. Gene Ontology (GO) and Kyoto Encyclopedia of Genes and Genomes (KEGG) pathway analysis of the hub genes were performed using WEB-based GEne SeT AnaLysis Toolkit (WebGestalt). The expression levels and survival analysis of hub genes were further assessed through Gene Expression Profiling Interactive Analysis (GEPIA) and the Kaplan-Meier plotter database. In total, 213 overlapping DEGs were identified, of which 109 were upregulated and 104 were downregulated. GO analysis revealed that the DEGs were predominantly involved in mitosis and cell division. KEGG pathways analysis demonstrated that most DEGs were significantly enriched in cell cycle pathway. GSEA revealed that the DEGs were mainly enriched in oocyte meiosis, cell cycle and DNA replication pathways. A key module was identified and 10 hub genes (*CENPF*, *KIF11*, *KIF23*, *TTK*, *MKI67*, *TOP2A*, *CDC45*, *MELK*, *AURKB*, and *BUB1*) were screened out. The expression and survival analysis disclosed that the 10 hub genes were upregulated in SS patients and could result in significantly reduced survival. Our study identified a series of metastasis-associated biomarkers involved in the progression of SS, and may provide novel therapeutic targets for SS metastasis.

## Introduction

Synovial sarcoma (SS) ranks the fourth most common form of soft tissue sarcoma (STS), comprising nearly 10% of total STSs worldwide. As an aggressive high-grade malignancy, SS predominantly affects children, adolescents and young adults. SS harbors the unique chromosomal translocation t(X;18) (p11.2; q11.2) which results in the formation of a fusion protein, *SS18-SSX* (Svejstrup, 2013). It has been demonstrated that the *SS18-SSX* fusion protein is the oncogenic driver in the development of SS (Nagai et al., 2001). And the underlying mechanism is considered to be that this fusion protein preferentially affects the cell growth, cell proliferation, cell invasion and metastasis, TP53 pathway, and chromatin remodeling mechanisms (Przybyl et al., 2012). Nowadays, radical surgery combined with radiotherapy and/or chemotherapy is the mainstay of therapy for localized SS (Desar et al., 2018). Despite the improvements in these treatments in the past two decades, about 49% of SS patients eventually develop local recurrence and/or distant metastasis (Krieg et al., 2011). Thus, it is urgent to identify the molecules that regulate SS metastasis, which would provide novel therapeutic targets for the treatment of SS.

To date, there are a variety of biomarkers considered to play important roles in SS metastasis, nevertheless, only few of them have been externally identified as statistically significant predictors of survival for SS patients, such as *SS18-SSX* (Sun et al., 2009), *P300* (Liu et al., 2019), *SHCBP1* (Peng et al., 2017), *VEGF* (Feng et al., 2018), *SUZ12* (Cho et al., 2018), *EDD1* (Cho et al., 2018), *EZH2* (Yalcinkaya et al., 2017), *FOXM1* (Maekawa et al., 2016), *IGFBP7* (Benassi et al., 2015), *MRP1* (Martin-Broto et al., 2014), *p27* (Kawauchi et al., 2001), *E-cadherin* (Saito et al., 2000), *HGF/c-MET* (Oda et al., 2000), etc. Moreover, the interactions among these biomarkers in SS are not investigated by integrated analysis. More importantly, the differential expression of metastasis-associated genes between SS patients with and without metastasis is not explored.

Based on bioinformatics analysis, the present study aimed at screening critical metastasis-associated biomarkers of SS, identifying hub genes in protein-protein interaction (PPI) networks, and evaluating their prognostic significance for predicting survival and metastasis in patients with SS. Two mRNA datasets (GSE40018 and GSE40021) were selected to screen the differentially expressed genes (DEGs) between metastasis SS samples and non-metastasis SS samples. To assess the underlying molecular mechanism that regulates SS metastasis, the DEGs were further analyzed by Gene Ontology (GO), Kyoto Encyclopedia of Genes and Genomes (KEGG) pathway, using the Database for Annotation, Visualization and Integrated Discovery (DAVID) and gene set enrichment analysis (GSEA). By constructing PPI network and using the Search Tool for the Retrieval of Interacting Genes (STRING) database and Cytoscape software, a key module was then screened out from the whole network, and based on the key module, the hub genes were identified. Subsequently, GO and KEGG pathway analysis of the identified hub genes were performed using the WEB-based GEne SeT AnaLysis Toolkit (WebGestalt) online tool. Finally, the expression and survival analysis of the hub genes were carried out by Gene Expression Profiling Interactive Analysis (GEPIA) and Kaplan-Meier plotter (KM plotter) online database, respectively. This study identified several potentially critical metastasis-associated biomarkers involved in the progress of SS, which may provide novel targets for anti-metastatic therapeutics in SS.

## Materials and Methods

### Microarray Data

The microarray expression data of GSE40018 and GSE40021 were obtained from the Gene Expression Omnibus database (GEO^1^). The dataset GSE40018 based on the platform of GPL13497 platform (Agilent-026652 Whole Human Genome Microarray 4 × 44K v2) including 17 non-metastasis SS samples and 17 metastasis SS samples. The dataset GSE40021 based on the platform of GPL6480 platform (Agilent-014850 Whole Human Genome Microarray 4 × 44K G4112F) containing 30 non-metastasis SS samples and 28 metastasis SS samples.

### DEGs Analysis

To screen the DEGs between non-metastasis SS samples and metastasis SS samples, we used the GEO2R online web tool^2^, which allows users to compare different gene expression data of two or more groups of samples. Adjusted *p*-value < 0.05 and | log (FC)| ≥ 1.0 were set as the thresholds for identifying DEGs. DEGs with logFC > 0 were considered as upregulated genes, and those with logFC < 0 were classified as downregulated genes. To identify the intersectional genes between GSE40018 and GSE40021, the Venny 2.1 online web tool^3^ was used to create a Venn diagram. Heatmaps of the DEGs were generated by HemI software (Version 1.0.3.7) (Deng et al., 2014), a toolkit for illustrating heatmaps.

### Go Functional and Pathway Enrichment Analyses

Based on the DAVID database^4^ (Version 6.8), we analyzed GO and KEGG pathway enrichment analyses for the DEGs. GO terms enrichment analysis were categorized into biological process (BP), cellular component (CC), and molecular function (MF). Benjamini-Hochberg false discovery rate (FDR) < 0.05 was specified for statistical significance.

### GSEA

GO and KEGG pathway enrichment analyses for the DEGs were further performed using GSEA 4.0 software, which was downloaded from GSEA home^5^ and run in a Java environment. The GSEA analysis was carried out as previously described (Subramanian et al., 2005). Samples from GSE40021 were classified into two groups as metastasis group and non-metastasis group, and meanwhile the DEGs were also separated into two groups as upregulated group and downregulated group. The number and type of permutations was defined as “1000” and “phenotype,” respectively. After that the c5.bp.v7.0.symbols.gmt, c5.cc.v7.0.symbols.gmt, c5.mf.v7.0.symbols.gmt and c2.cp.kegg.v7.0.symbols.gmt downloaded from the Molecular Signatures Database (MSigDB) (Liberzon et al., 2015) were chosen as the reference gene sets to perform GSEA analysis. The cut-off criteria were set as nominal *P* < 0.05, FDR *q*-value < 0.25 and enrichment score (ES) > 0.6.

### PPI Network Construction and Module Analysis

The STRING database^6^ was used to characterize the PPI network of DEGs, and the combined score > 0.4 was used as the cut-off criteria. The PPI network was then mapped using Cytoscape software (Version 3.7.2). In order to screen the most significant module of PPI network, the Molecular Complex Detection (MCODE) plugin (Version 1.6.1) in Cytoscape was carried out with MCODE scores > 5, degree cut-off = 2, node score cut-off = 0.2, *k*-core = 2 and max. depth = 100 (Bader and Hogue, 2003). After that, GO functional analysis of DEGs in the most significant module was performed using Cytoscape ClueGO (Version 2.5.7) and CluePedia (Version 1.5.7) plugins with a threshold value of FDR < 0.05 and κ-coefficient of 0.4 (Bindea et al., 2009, 2013).

### Hub Genes Selection and Analysis

The hub genes were selected from the above most significant module network using the Cytoscape CytoHubba plugin (Version 0.1). Genes with a degree ≥ 10 were defined as hub genes. Also, if an adjusted *p*-value < 0.05, the selection of the hub genes is considered statistically significant. Here, the genes with the top 10 highest degree values were considered as real hub genes. Meanwhile, GO and KEGG pathway enrichment analyses were carried out for the top 10 hub genes using the WebGestalt^7^ with a threshold of FDR < 0.05.

### Survival Analysis

The comparison of the identified hub genes expression between tumor and normal samples of SS was analyzed through GEPIA^8^ (Tang et al., 2017), a public database for cancer and normal gene expression profiling and interactive analyses. A difference or result with *p*-value < 0.05 and |Log_2_FC| > 1 was regarded as statistically significant. The prognostic values of these hub genes for SS patients were further validated through KM plotter database^9^. Database including 259 patients with relapse-free and overall survival information was used for validation. Briefly, patients were categorized into two groups (high expression and low expression) according to the median of each hub gene expression. Subsequently, the identified hub genes were imported into the database, and the Kaplan-Meier survival plots were then generated and the hazard ratio (HR) and its associated 95% confidence intervals (CI) and log rank test *P* value were calculated. Log-rank test results with *P* < 0.05 were considered as statistically significant.

## Results

### Identification of DEGs

**FIGURE 1:**
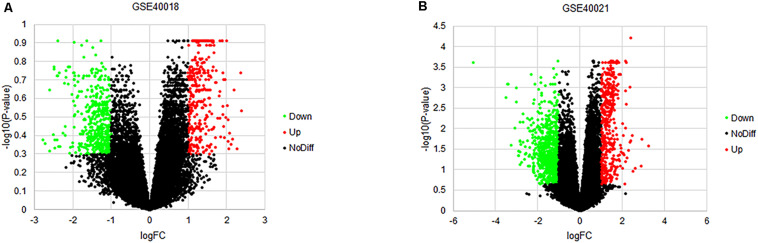
Volcano plots of DEGs detected from the datasets of GSE40018 and GSE40021. **(A)** GSE40018, **(B)** GSE40021. The red dots represent upregulated DEGs; the green dots mean downregulated DEGs; the black dots denote no differentially expressed genes.

**FIGURE 2:**
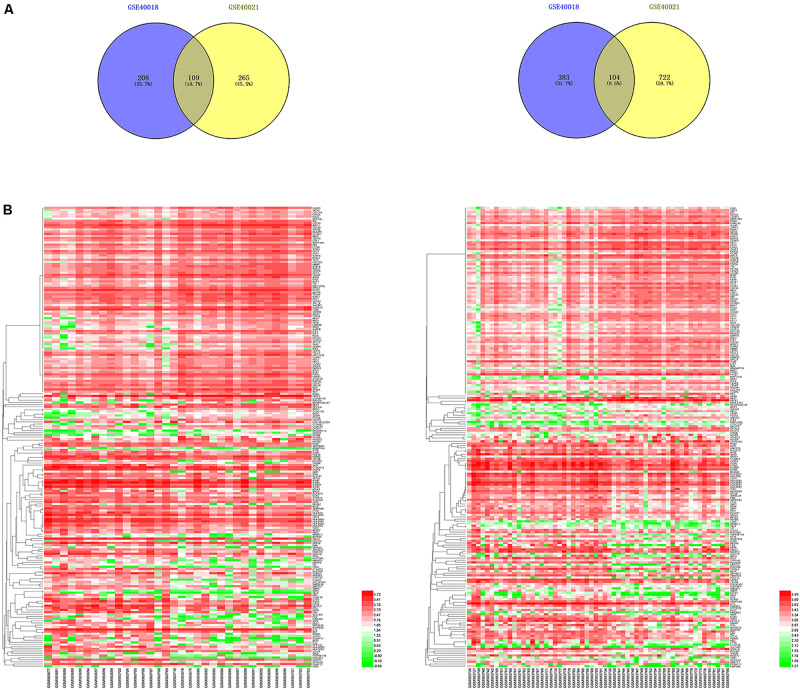
Identification of DEGs associated with SS metastasis. **(A)** Venn diagram demonstrates the crossed genes shared by GSE40018 and GSE40021 datasets. Left panel represents the upregulated co-differentially expressed genes between GSE40018 and GSE40021 datasets, whereas the right panel represents the downregulated intersectional genes between the two datasets. **(B)** Heatmap of the top 213 DEGs associated with SS metastasis in the two datasets. Left, GSE40018; right, GSE40021.

By using the GEO2R online tool from the GEO, we found that there were 804 DEGs (317 upregulated and 487 downregulated) in GSE40018, 1200 DEGs (374 upregulated and 826 downregulated) in GSE40021, which were differentially expressed between metastasis SS samples and non-metastasis SS samples as shown by volcano plots in Figures 1A,B. Further analysis of these DEGs by using Venn diagram, we found that there were 213 overlapping DEGs including 109 upregulated and 104 downregulated genes between the two datasets (Figure 2A; Table 1), which were identified according to the cut-off criteria (adjusted *p*-value < 0.05 and | logFC| ≥ 1). Meanwhile, the 213 overlapping DEGs in GSE40018 and GSE40021 were displayed in a heatmap (Figure 2B).

**TABLE 1:** Identification of DEGs associated with SS metastasis.

Regulation	Genes
Upregulated (*n* = 109)	*KNL1, ORC6, DTL, CDCA5, CEP55, KIAA0101, GINS2, BIRC5, TOP2A, BUB1, CLSPN, AURKB, AURKA, MND1, KIFC1, AGMO, CDK1, MYBL2, KIF4A, TROAP, FOXM1, PLK4, MEST, BRIP1, AUNIP, DIAPH3, BUB1B, PTTG2, PRC1, FANCA, UHRF1, TACC3, POLQ, EXO1, HIST1H1B, GALNT9, CENPE, CENPU, KIF20A, TTK, HOXB9, FAM83D, ESPL1, DPYSL4, TICRR, NEK2, ANLN, E2F8, HIST1H2AL, CDCA8, HOXD10, CDCA2, CKAP2L, E2F2, PIF1, HOXD9, KIF23, TYMS, NDC80, CENPO, SPC25, ASPM, CDC45, GTSE1, CCNA2, CCNB2, GSG2, TPX2, HMMR, CDC25C, RAD54L, NUF2, PTTG1, KIF15, DLGAP5, NR2F2, BLM, RAD51, SPAG5, MELK, ERCC6L, RAD51AP1, SAPCD2, CCDC150, CDCA3, RRM2, SGO1, KIF11, ZNF695, ARHGAP11A, SPC24, SKA1, ESCO2, MKI67, CDC25A, CENPM, LPCAT1, CENPA, CENPI, NR2F2-AS1, CENPF, ZFHX4-AS1, FOXN4, ATG9B, E2F7, DKFZP434L187, PBK, FANCI, SHCBP1*
Downregulated (*n* = 104)	*ROM1, DIO3, ADAMTSL4, ACKR1, PLA2G16, PLA2G4A, HDC, CYP11A1, SELP, SERPING1, CTSG, ACP5, FBLN2, S100A16, FGG, ICAM1, MAMDC2, ZMYND12, ADIRF, NAALAD2, HSPB2, ASPA, SCRG1, ITM2A, MS4A6A, RNASE4, ADGRG1, TMEM246, NFKBIZ, PPP2R2B, SERPINB1, PID1, PERP, CCL14, C1S, ECM2, PHYHD1, HLA-DPA1, IL33, ADRA1B, MYOF, CASP4, MET, CTSZ, ANKRD29, GSAP, HLA-DRB5, PLA2G4C, SLC18A2, GBP1, IFIH1, PLSCR1, GPAT3, CD74, TNFRSF11B, SMPDL3A, MFSD7, IFITM1, PDE4B, MSLN, HLA-DQB1, FAM107A, TCEAL2, GBP2, IFITM3, CD69, TLR2, C1orf186, SELE, MEDAG, HS6ST2, KITLG, DPYSL3, IFITM4P, HLA-DPB1, PARM1, CRB1, CRYAB, NMNAT3, GMPR, GOLT1A, IFITM2, HLA-DPB2, HLA-DRB1, NT5E, SP100, WFIKKN2, ADGRG6, EMB, PTN, CFI, THEM4, APOL6, PCDH11Y, DMRTC1, UBXN10, DLG2, HLA-DOA, NUDT9P1, PQLC2L, BEX5, FAM19A4, FGF14-AS2, HLA-DRA*

### Functional Enrichment Analysis of DEGs

**FIGURE 3:**
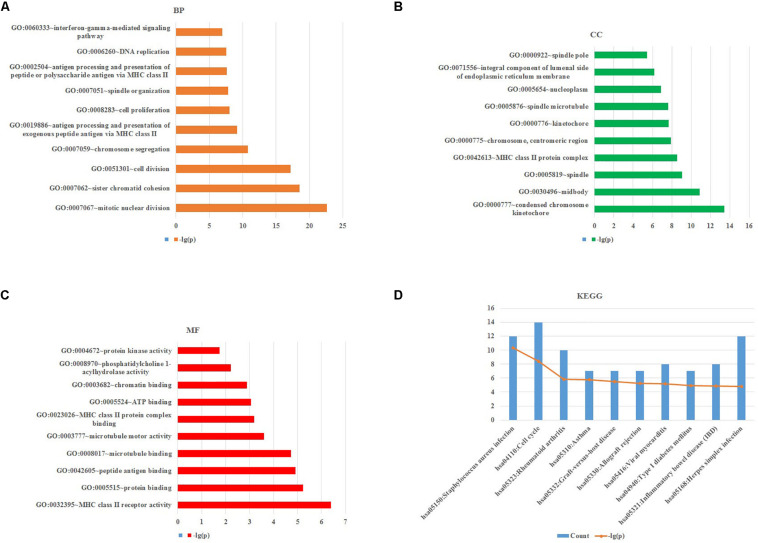
GO and KEGG pathway enrichment analyses of DEGs using the DAVID database. **(A)** Biological processes, **(B)** cellular components, **(C)** molecular functions, **(D)** the signaling pathways from the GSE40018 and GSE40021 datasets.

To obtain further investigation of the functions of identified 213 overlapping DEGs, we used the DAVID database to perform GO and KEGG pathway enrichment analyses of DEGs. It was found that according to biological process, the identified 213 overlapping DEGs were mainly enriched in mitotic nuclear division, sister chromatid cohesion and cell division (Figure 3A; Table 2). For cellular component, it was uncovered that most DEGs were enriched in condensed chromosome kinetochore, midbody and spindle (Figure 3B; Table 2). In terms of molecular function, it was revealed that DEGs were mainly enriched in MHC class II receptor activity, protein binding and peptide antigen binding (Figure 3C; Table 2). For KEGG pathway analysis, significantly enriched pathways of DEGs were enriched in staphylococcus aureus infection, cell cycle and rheumatoid arthritis signaling pathways (Figure 3D; Table 3).

**TABLE 2:** The top 3 enriched GO terms of DEGs associated with SS metastasis.

Category	Term	Count	FDR
OTERM_BP_DIRECT	GO:0007067∼mitotic nuclear division	32	3.91E–20
GOTERM_BP_DIRECT	GO:0007062∼sister chromatid cohesion	21	4.52E–16
GOTERM_BP_DIRECT	GO:0051301∼cell division	31	1.07E–14
GOTERM_CC_DIRECT	GO:0000777∼condensed chromosome kinetochore	16	4.63E–11
GOTERM_CC_DIRECT	GO:0030496∼midbody	16	1.75E–08
GOTERM_CC_DIRECT	GO:0005819∼spindle	14	1.14E–06
GOTERM_MF_DIRECT	GO:0032395∼MHC class II receptor activity	6	5.53E–04
GOTERM_MF_DIRECT	GO:0005515∼protein binding	127	0.008032
GOTERM_MF_DIRECT	GO:0042605∼peptide antigen binding	6	0.016106

**TABLE 3:** The top 3 enriched KEGG pathways of DEGs associated with SS metastasis.

Category	Term	Count	FDR
KEGG_PATHWAY	hsa05150:Staphylococcus aureus infection	12	4.98E–08
KEGG_PATHWAY	hsa04110:Cell cycle	14	4.36E–06
KEGG_PATHWAY	hsa05323:Rheumatoid arthritis	10	0.001609

### GSEA Analysis

Based on GO and KEGG pathway enrichment analyses of DEGs, GSEA software was also used to identify the potential molecular mechanisms underlying SS metastasis. In agreement with the above results of DAVID, GSEA results indicated that DEGs were significantly involved in GO_ SISTER_CHROMATID_SEGREGATION, GO_MITOTIC_SPINDLE_ORGANIZATION and GO_ CHROMOSOME_SEGREGATION (BP) (Supplementary Figure S1A); and GO_CONDENSED_CHROMOSOME, GO_KINETOCHORE and GO_CHROMOSOME_CENTRO-MERIC_REGION (CC) (Supplementary Figure S1B); and GO_DNA_DIRECTED_DNA_POLYMERASE_ACTIVITY, GO_ SINGLE_STRANDED_DNA_BINDING and GO_N_ACYLTR-ANSFERASE_ACTIVITY (MF) (Supplementary Figure S1C). For KEGG pathway, significantly enriched pathways of DEGs were enriched in oocyte meiosis, cell cycle and DNA replication signaling pathways (Supplementary Figure S1D).

### PPI Network and Module Analysis

**FIGURE 4:**
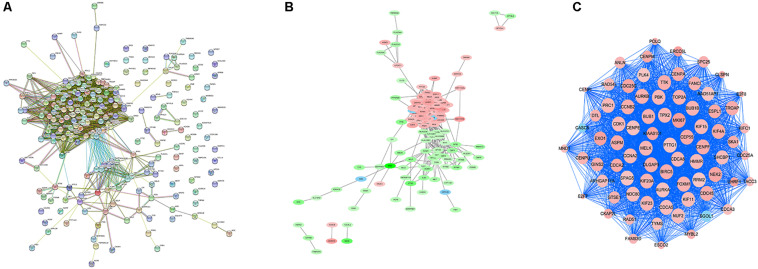
PPI network analysis and identification of hub genes. **(A)** Protein-protein interactions of DEGs were analyzed using the STRING database. **(B)** The PPI network of DEGs was established using Cytoscape software. The red nodes indicated upregulated genes, whereas the green nodes represented downregulated genes. **(C)** The most significant module in the PPI network was identified using MCODE plugin. The size of the node represented the node degree. Nodes with higher the degree represented more bigger size.

**FIGURE 5:**
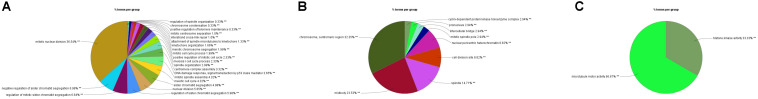
GO analyses of DEGs in the most significant module using Cytoscape plugins ClueGO and CluePedia. **(A)** Percentages of biological processes terms per group. **(B)** Percentages of cellular components terms per group. **(C)** Percentages of molecular functions terms per group.

In order to explore the interaction between the screened 213 DEGs, we used the STRING database to construct the PPI network. As shown in Figure 4A, a relevant PPI network was successfully constructed, which contained 204 nodes and 3210 edges. Then, this PPI network was visualized using Cytoscape software (Figure 4B). Module analysis was then conducted using the MCODE plugin of Cytoscape based on the whole network. As shown in Figure 4C, the identified key module from the whole network was detected with 77 nodes and 2650 edges. Next, the DEGs in this key module were then subjected to perform GO and KEGG pathway enrichment analyses using Cytoscape plugins ClueGO and CluePedia, and the results revealed that most DEGs were significantly enriched in mitotic nuclear division 36.54%, negative regulation of sister chromatid segregation 6.98%, and regulation of mitotic sister chromatid segregation 6.64% for BP (Figure 5A; Supplementary Figure S2A); and chromosome, centromeric region 32.35%, midbody 23.53%, and spindle 14.71% for CC (Figure 5B; Supplementary Figure S2B); and microtubule motor activity 66.67% and histone kinase activity 33.33% for MF (Figure 5C; Supplementary Figure S2C).

### Hub Genes Selection and Survival Analysis

**FIGURE 6:**
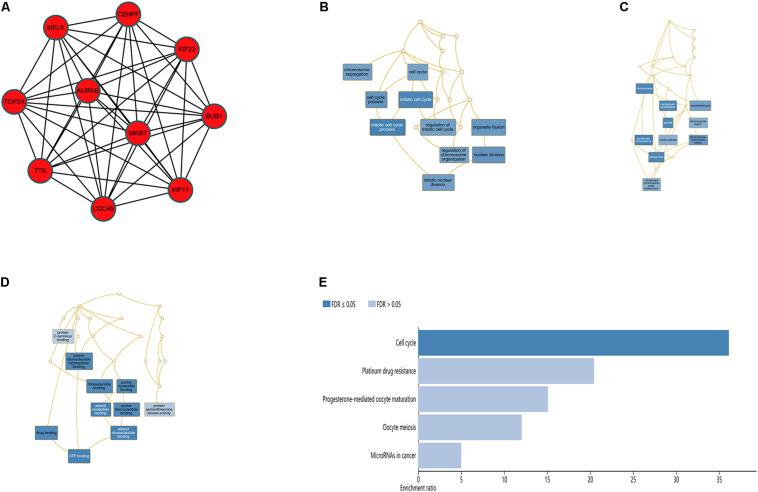
Significantly enriched GO and KEGG pathways for the 10 hub genes using the WebGestalt database. **(A)** Ten hub genes were identified from the most significant module. **(B)** Biological process, **(C)** cellular component, **(D)** molecular function, **(E)** KEGG pathways for the 10 hub genes.

The top 10 hub genes were identified using degrees > 10. The top 10 hub genes were filtered out from the key module using the CytoHubba plugin, including *CENPF*, *KIF11*, *KIF23*, *TTK*, *MKI67*, *TOP2A*, *CDC45*, *MELK*, *AURKB*, and *BUB1* (Figure 6A; Table 4). Then, the online tool WebGestalt was employed to further discover the GO and KEGG pathway enrichment analyses for the identified 10 hub genes. It was found that the 10 hub genes were significantly enriched in mitotic cell cycle process, cellular component organization, nuclear division, cell cycle process, and organelle fission for BP (Figure 6B; Supplementary Table S1); and condensed chromosome, chromosome, spindle, microtubule cytoskeleton, and kinetochore for CC (Figure 6C; Supplementary Table S2); and ATP binding, adenyl ribonucleotide binding, adenyl nucleotide binding, drug binding, and purine ribonucleoside triphosphate binding for MF (Figure 6D; Supplementary Table S3). In terms of KEGG pathway, the 10 hub genes were significantly enriched in cell cycle (Figure 6E; Supplementary Table S4).

**TABLE 4:** Hub genes identified in the key module.

Genes	Description	Degree	adj. *P*-value	Log FC
*CENPF*	Centromere Protein F	81	3.40E–04	1.56338
*KIF11*	Kinesin Family Member 11	90	0.001198	1.244453
*KIF23*	Kinesin Family Member 23	88	4.73E–04	1.355373
*TTK*	TTK Protein Kinase	84	0.001272	1.507303
*MKI67*	Proliferation marker protein Ki-67	79	4.74E–04	1.835864
*TOP2A*	DNA topoisomerase II alpha	82	0.002458	1.434285
*CDC45*	Cell division control protein 45	84	4.74E–04	1.55918
*MELK*	Maternal embryonic leucine zipper kinase	80	0.003088	1.351395
*AURKB*	Aurora Kinase B	83	0.003495	1.450697
*BUB1*	BUB1 mitotic checkpoint serine/threonine kinase	85	9.10E–04	1.393907

**FIGURE 7:**
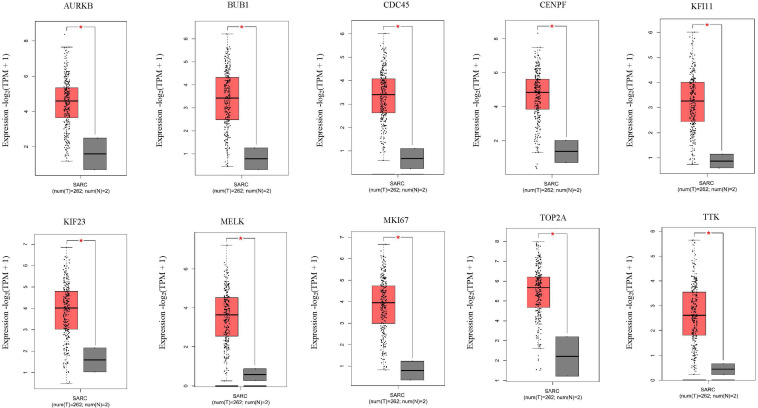
Validation of the 10 hub genes overexpression in SS tissues using the GEPIA database. *P < 0.05, unpaired Student’s *t*-test.

**FIGURE 8:**
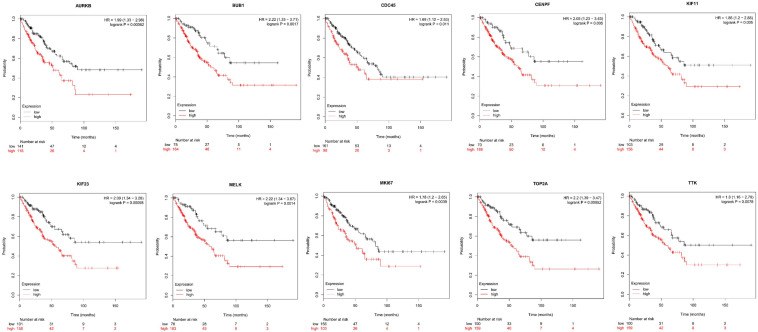
Kaplan Meier curves for overall survival analysis of the 10 hub genes in SS patients. Red line represented the high expression group, whereas black line denoted the low expression group.

**FIGURE 9:**
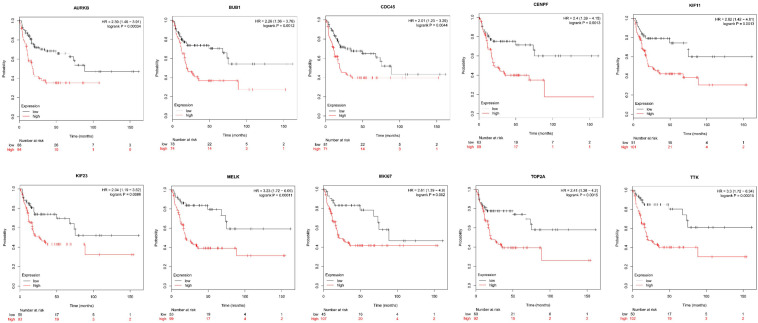
Kaplan Meier curves for recurrence-free survival analysis of the 10 hub genes in SS patients. Red line represented the high expression group, whereas black line denoted the low expression group.

Next, comparing the expression levels of hub genes was performed using GEPIA database. It was found that the 10 hub genes displayed significantly higher expression in SS patients compared to the normal control subjects (Figure 7). Thereafter, the effects of 10 hub genes on overall survival were conducted using Kaplan Meier plots. As shown in Figure 8, SS patients with high expression of *CENPF*, *KIF11*, *KIF23*, *TTK*, *MKI67*, *TOP2A*, *CDC45*, *MELK*, *AURKB*, and *BUB1* had worse overall survival. Moreover, SS patients with high expression of *CENPF*, *KIF11*, *KIF23*, *TTK*, *MKI67*, *TOP2A*, *CDC45*, *MELK*, *AURKB*, and *BUB1* predicted worse recurrence-free survival (Figure 9).

## Discussion

Here, we identified 213 DEGs that were associated with SS metastasis from the GSE40018 and GSE40021 datasets, including 109 upregulated and 104 downregulated genes. According to GO and KEGG pathway enrichment analyses of the 213 DEGs, DEGs were found to be significantly enriched in mitosis, cell division and cell cycle pathway. Through a PPI network, we identified a key module containing 77 nodes and 2650 edges. More importantly, based on this key module, we screened 10 hub genes, which were further demonstrated to be closely related to the progression and prognosis of SS.

Hub genes, namely *CENPF, KIF11, KIF23, TTK, MKI67, TOP2A, CDC45, MELK, AURKB* and *BUB1*, were screened based on the PPI network of DEGs, suggesting the 10 hub genes may play key roles in SS metastasis. As a cell cycle-associated nuclear antigen, *CENPF* is involved in chromosome segregation during mitosis, and found to be related to tumor growth in many human malignancies. *CENPF* has been reported to be frequently expressed at high levels in hepatocellular carcinoma, and suppression of *CENPF* leads to growth inhibition and cell cycle arrest (Dai et al., 2013). Knockdown of *CENPF* attenuates the cell growth and invasion of gastric cancer (Chen et al., 2019). Increased expression of *CENPF* in prostate cancer suggests poor prognosis of patients (Zhuo et al., 2015). Furthermore, *CENPF* collaborates with *FOXM1* to synergistically induce target gene expression and leads to activation of crucial signaling pathways correlated with tumor malignancy (Aytes et al., 2014).

*KIF11*, belonging to the kinesin-5 family, is involved in the tetrameric microtubule cross-linkage, cell mitosis, the cell cycle, and differentiation (Roostalu et al., 2011). Studies have shown that *KIF11* is overexpressed in many cancers, such as gastric cancer (Imai et al., 2017), breast cancer (Zhou et al., 2019), oral cancer (Daigo et al., 2018), renal cell (Sun et al., 2013), and astrocytic cancers (Liu et al., 2016). Similarly, *KIF23*, as a key regulator of cytokinesis, is also found to be correlated with poor prognosis for patients with hepatocellular carcinoma (Sun et al., 2015), glioma (Sun et al., 2016), gastric cancer (Li et al., 2019), and non-small-cell lung cancer (Vikberg et al., 2017).

Previous studies have revealed that *TTK* is highly expressed in several malignant tumors, and its increased expression indicates a poor prognosis. *TTK* contributes to the proliferation and invasion of tumor cells via regulating the mitotic process (Yang et al., 2010). *MKI67*, also known as *Ki-67*, acts as a biological surfactant to disperse mitotic chromosomes (Cuylen et al., 2016), which has been demonstrated in various carcinomas, including gastric, esophageal, colonic, rectal, and esophageal squamous cell carcinoma (Volkweis et al., 2012). Currently, a high *MKI67* expression predicts for poor prognosis in patients with retroperitoneal soft tissue sarcomas (Morizawa et al., 2016). *TOP2A* gene, residing on chromosome 17 (17q21-q22), can regulate DNA replication, chromosome segregation, and cell cycle progression (Zeng et al., 2019). An elevated *TOP2A* expression is observed in many tumor tissues, and significantly associated with *MKI67* expression as well as tumor aggressiveness and poor outcome (Brase et al., 2010).

Numerous evidences have shown that *CDC45* is required during the process of DNA replication, and *CDC45* overexpression is also associated with cancer cell proliferation (Pollok et al., 2007). *MELK*, as a member of kinases family which is directly regulated by the *FOXM1* transcription factor, has been proven to function as an oncogenic gene that is closely associated with mitotic progression as well as the proliferation in multiple human cancers (Wang et al., 2014, 2016). *AURKB* is highly expressed in many cancers. By regulating the cell cycle progression and mitosis, *AURKB* is thus involved in tumorigenesis and predicts poor prognosis in numerous human tumors (Wan et al., 2019). *BUB1*, a mitotic checkpoint serine/threonine kinase, regulates chromosome segregation, and indicates a poor clinical outcome in many cancers (Han et al., 2015). Taken together, our data revealed that almost all the 10 hub genes were correlated with cell cycle and mitosis.

Although the 10 hub genes have critical roles in the process of a variety of tumors, they are not reported to participate in SS progression. Based on the functions of the above identified 10 hub genes, it is established that the 10 hub genes promote tumor progression mainly by regulating the cell cycle or chromatin replication. Recent studies have showed that in the process of carcinogenesis, the cell cycle is critical and dysregulated cell cycle may cause uncontrolled cell proliferation, survival and differentiation which are essential for the early stages of carcinogenesis (Scott et al., 2015). Consisted with the above researches, our data from GO and KEGG enrichment as well as GSEA analyses indicated that most DEGs were involved in cell cycle, thus supporting the contribution of cell cycle to SS progression and metastasis. In order to further validate our results, the expression levels and the survival analysis of the 10 hub genes in SS were assessed using GEPIA and the Kaplan-Meier plotter database. As expected, all these hub gens were significantly higher in SS samples compared to normal samples, suggesting their crucial roles in carcinogenesis. More importantly, in agreement with the above reports, the survival analysis revealed that the 10 hub genes also had high prognostic values for SS, which would provide novel prognostic biomarkers for therapeutic targets for SS treatment.

Metastasis is a complex multistep process, characterized by a high rate of local invasion and early distant metastasis (Ren et al., 2017). Genetic and epigenetic alterations that occur in tumor cells are likely to contribute to tumor cell invasion and metastasis (Gibson et al., 2016). Recent evidences have revealed a key role of cell cycle arrest in cancer invasion and metastasis. Iwasaki et al. (1995) demonstrated that hepatocellular carcinoma cells acquire the ability to metastasize in the G1 phase of the cell cycle. In prostate cancer, by inhibiting the G1-to-S phase transition of the cell cycle, Runx2 promotes the invasiveness and bone metastasis (Baniwal et al., 2010). Also, the significant relationship between invasive cells and the G1/G0 cell cycle state is observed in breast cancer metastasis (Qian et al., 2013). However, to date, there were few researches regarding the effect of cell cycle on tumor cell metastasis in SS. Herein, this study reported that the identified 10 hub genes were mostly enriched in cell cycle regulation, indicating their roles as prognostic markers in SS metastasis.

In our study, a major limitation is that the expression and the survival analyses of the identified 10 hub genes were validated not in synovial sarcoma specifically, but for all sarcomas (GEPIA dataset does not have subdivisions into sarcoma types). However, despite this limitation, considering the data of GEPIA are from The Cancer Genome Atlas (TCGA), and because the expression of the 10 hub genes in Sarcoma-TCGA dataset is higher than normal tissues (data not shown), the results are still significant. For survival analysis, because only nine SS tissue samples were found in Sarcoma-TCGA dataset, the sample size is too small to supply meaningful statistical results. For this reason, the survival analyses were performed in all sarcoma dataset. When the amount of available SS samples will become large enough, the survival analyses of the identified 10 hub genes should be performed. Therefore, additional confirmation of our findings should be reserved for future studies.

## Conclusion

In summary, based on the bioinformatics analysis of DEGs in SS metastasis samples and non-metastasis samples, this research successfully identified 10 hub genes (*CENPF*, *KIF11*, *KIF23*, *TTK*, *MKI67*, *TOP2A*, *CDC45*, *MELK*, *AURKB*, and *BUB1*). The critical pathway enriched in the 10 hub genes was cell cycle, which would uncover mechanistic insights into SS metastasis, thus promoting the development for the treatment of SS.

## Data Availability Statement

Publicly available datasets were analyzed in this study, these can be found in the Gene Expression Omnibus database (GEO, https://www.ncbi.nlm.nih.gov/geo/) (GSE40018 and GSE40021).

## Author Contributions

YS and CP conceived and designed the study. YS wrote the manuscript. CP and GC revised and polished the manuscript. XL, FW, and XW performed the analysis and interpretation of data. All authors read and approved the final manuscript.

## Conflict of Interest

The authors declare that the research was conducted in the absence of any commercial or financial relationships that could be construed as a potential conflict of interest.

## Footnotes

1 https://www.ncbi.nlm.nih.gov/geo/

2 http://www.ncbi.nlm.nih.gov/geo/geo2r

3 http://bioinfogp.cnb.csic.es/tools/venny/

4 https://david.ncifcrf.gov/

5 https://www.gsea-msigdb.org/gsea/index.jsp

6 https://string-db.org/

7 http://www.webgestalt.org/

8 http://gepia.cancer-pku.cn/index.html

9 http://kmplot.com/

## Supplementary Material

FIGURE S1 GSEA analysis of DEGs in GSE40021 dataset. **(A)** Biological processes, **(B)** cellular components, **(C)** molecular functions, **(D)** the signaling pathways.

FIGURE S2 Function annotation of DEGs in the most significant module using Cytoscape plugins ClueGO and CluePedia. **(A)** Biological processes, **(B)** cellular components, **(C)** molecular functions. Different node colors represented different functional groups.
