# Supplementary figures and images for: Identification of Metastasis-Associated Biomarkers in Synovial Sarcoma Using Bioinformatics Analysis
Source: Front Genet. 2020 Sep 11;11:530892. doi: 10.3389/fgene.2020.530892 (PMC7518102; doi:10.3389/fgene.2020.530892)

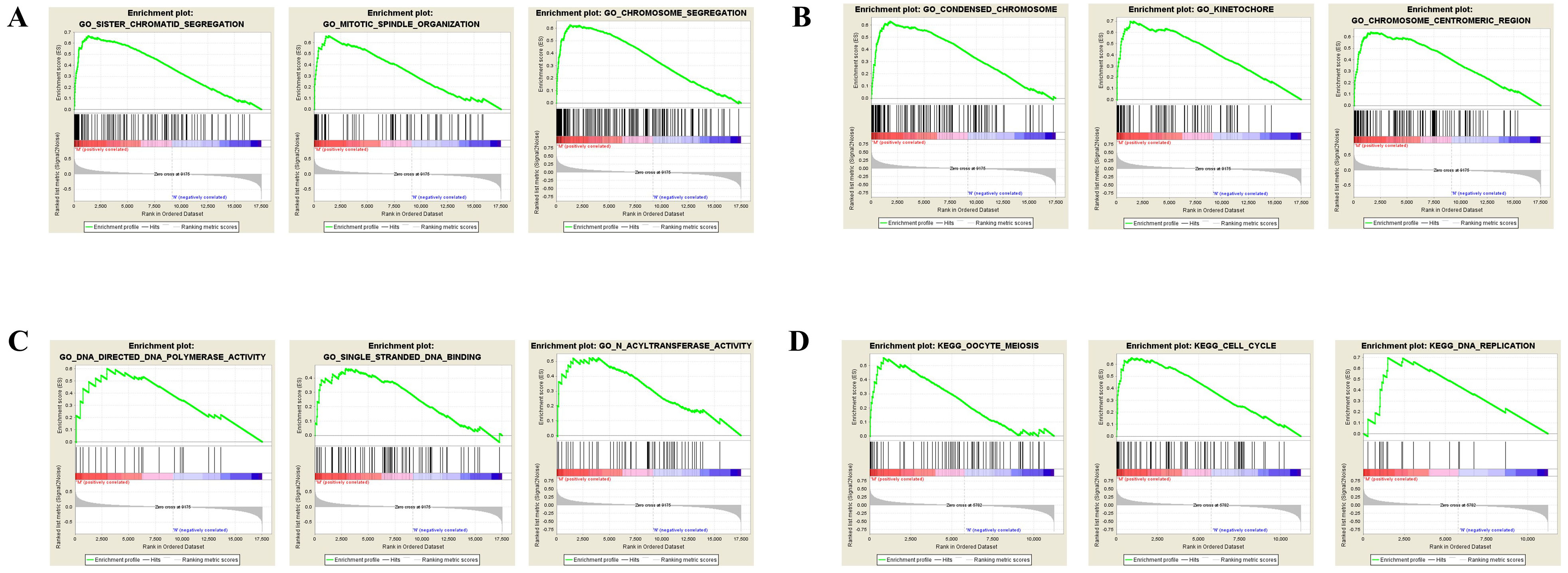

Supplement: FIGURE S1 — GSEA analysis of DEGs in GSE40021 dataset. (A) Biological processes, (B) cellular components, (C) molecular functions, (D) the signaling pathways. [file Image_1.JPEG]
