# Supplementary material for: Identification of Metastasis-Associated Biomarkers in Synovial Sarcoma Using Bioinformatics Analysis
Source: Front Genet. 2020 Sep 11;11:530892. doi: 10.3389/fgene.2020.530892 (PMC7518102; doi:10.3389/fgene.2020.530892)
Supplement: Supplementary file 3 [file Data_Sheet_1.docx]

**Supplementary Table S1 List of GO terms of Biological Processes (BP) of the 10 hub genes obtained via WebGestalt**

| **GeneSet** | **Description** | **FDR** | **Genes** |
| --- | --- | --- | --- |
| GO:1903047 | mitotic cell cycle process | 4.80E-10 | *CENPF;KIF11;KIF23;TTK;MKI67;TOP2A;CDC45;MELK;AURKB;BUB1* |
| GO:0000278 | mitotic cell cycle | 1.23E-09 | *CENPF;KIF11;KIF23;TTK;MKI67;TOP2A;CDC45;MELK;AURKB;BUB1* |
| GO:0000280 | nuclear division | 1.50E-08 | *CENPF;KIF11;KIF23;TTK;MKI67;TOP2A;AURKB;BUB1* |
| GO:0022402 | cell cycle process | 1.50E-08 | *CENPF;KIF11;KIF23;TTK;MKI67;TOP2A;CDC45;MELK;AURKB;BUB1* |
| GO:0048285 | organelle fission | 2.43E-08 | *CENPF;KIF11;KIF23;TTK;MKI67;TOP2A;AURKB;BUB1* |
| GO:0140014 | mitotic nuclear division | 6.42E-08 | *CENPF;KIF11;KIF23;TTK;MKI67;AURKB;BUB1* |
| GO:0007346 | regulation of mitotic cell cycle | 9.80E-08 | *CENPF;KIF11;TTK;MKI67;TOP2A;CDC45;AURKB;BUB1* |
| GO:0007059 | chromosome segregation | 9.80E-08 | *CENPF;KIF23;TTK;MKI67;TOP2A;AURKB;BUB1* |
| GO:0033044 | regulation of chromosome organization | 1.26E-07 | *CENPF;TTK;MKI67;TOP2A;CDC45;AURKB;BUB1* |
| GO:0007049 | cell cycle | 1.36E-07 | *CENPF;KIF11;KIF23;TTK;MKI67;TOP2A;CDC45;MELK;AURKB;BUB1* |

**Supplementary Table S2 List of GO terms of Cellular Component (CC) of the 10 hub genes obtained via WebGestalt**

| **GeneSet** | **Description** | **FDR** | **Genes** |
| --- | --- | --- | --- |
| GO:0000793 | condensed chromosome | 8.79E-05 | *CENPF;MKI67;TOP2A;AURKB;BUB1* |
| GO:0005694 | chromosome | 1.25E-04 | *CENPF;TTK;MKI67;TOP2A;CDC45;AURKB;BUB1* |
| GO:0005819 | spindle | 1.49E-04 | *CENPF;KIF11;KIF23;TTK;AURKB* |
| GO:0015630 | microtubule cytoskeleton | 1.49E-04 | *CENPF;KIF11;KIF23;TTK;TOP2A;CDC45;AURKB* |
| GO:0000776 | kinetochore | 1.49E-04 | *CENPF;TTK;AURKB;BUB1* |
| GO:0000775 | chromosome, centromeric region | 5.59E-04 | *CENPF;TTK;AURKB;BUB1* |
| GO:0044430 | cytoskeletal part | 8.72E-04 | *CENPF;KIF11;KIF23;TTK;TOP2A;CDC45;AURKB* |
| GO:0000940 | condensed chromosome outer kinetochore | 0.0026 | *CENPF;BUB1* |
| GO:0072686 | mitotic spindle | 0.0026 | *KIF11;KIF23;AURKB* |

**Supplementary Table S3 List of GO terms of Molecular Function (MF) of the 10 hub genes obtained via WebGestalt**

| **GeneSet** | **Description** | **FDR** | **Genes** |
| --- | --- | --- | --- |
| GO:0005524 | ATP binding | 1.13E-04 | *KIF11;KIF23;TTK;MKI67;TOP2A;MELK;AURKB;BUB1* |
| GO:0032559 | adenyl ribonucleotide binding | 1.13E-04 | *KIF11;KIF23;TTK;MKI67;TOP2A;MELK;AURKB;BUB1* |
| GO:0030554 | adenyl nucleotide binding | 1.13E-04 | *KIF11;KIF23;TTK;MKI67;TOP2A;MELK;AURKB;BUB1* |
| GO:0008144 | drug binding | 2.07E-04 | *KIF11;KIF23;TTK;MKI67;TOP2A;MELK;AURKB;BUB1* |
| GO:0035639 | purine ribonucleoside triphosphate binding | 2.07E-04 | *KIF11;KIF23;TTK;MKI67;TOP2A;MELK;AURKB;BUB1* |
| GO:0032555 | purine ribonucleotide binding | 2.07E-04 | *KIF11;KIF23;TTK;MKI67;TOP2A;MELK;AURKB;BUB1* |
| GO:0017076 | purine nucleotide binding | 2.07E-04 | *KIF11;KIF23;TTK;MKI67;TOP2A;MELK;AURKB;BUB1* |
| GO:0032553 | ribonucleotide binding | 2.07E-04 | *KIF11;KIF23;TTK;MKI67;TOP2A;MELK;AURKB;BUB1* |
| GO:0004674 | protein serine/threonine kinase activity | 0.019988 | *TTK;MELK;AURKB;BUB1* |
| GO:0008022 | protein C-terminus binding | 0.029521 | *CENPF;MKI67;TOP2A* |

**Supplementary Table S4 List of enriched KEGG pathways of the 10 hub genes obtained via WebGestalt**

| **GeneSet** | **Description** | **FDR** | **Genes** |
| --- | --- | --- | --- |
| hsa04110 | Cell cycle | 0.014213 | *TTK;CDC45;BUB1* |
| hsa01524 | Platinum drug resistance | 1 | *TOP2A* |
| hsa04914 | Progesterone-mediated oocyte maturation | 1 | *BUB1* |
| hsa04114 | Oocyte meiosis | 1 | *BUB1* |
| hsa05206 | MicroRNAs in cancer | 1 | *KIF23* |
